# Sugarcane Bagasse as an Efficient Biosorbent for Methylene Blue Removal: Kinetics, Isotherms and Thermodynamics

**DOI:** 10.3390/ijerph17020526

**Published:** 2020-01-14

**Authors:** Thaisa Caroline Andrade Siqueira, Isabella Zanette da Silva, Andressa Jenifer Rubio, Rosângela Bergamasco, Francielli Gasparotto, Edneia Aparecida de Souza Paccola, Natália Ueda Yamaguchi

**Affiliations:** 1 Centro Universitário de Maringá—Unicesumar, Maringá 87050-900, Brazil; thaisasiqueira@outlook.com (T.C.A.S.); isab.zanette@hotmail.com (I.Z.d.S.); 2 Programa de Pós-Graduação em Tecnologias Limpas—Unicesumar, Instituto Cesumar de Ciência, Tecnologia e Inovação—ICETI, Maringá 87050-900, Brazil; andressajrubio@gmail.com (A.J.R.); francielli.gasparotto@unicesumar.edu.br (F.G.); edneia.paccola@unicesumar.edu.br (E.A.d.S.P.); 3 Programa de Pós-Graduação em Engenharia Química, Departamento de Engenharia Química, Universidade Estadual de Maringá, Maringá 87020-900, Brazil; rosagela@deq.uem.br

**Keywords:** adsorption, methylene blue, sugarcane bagasse, sustainable, wastewater

## Abstract

Adsorption in biomass has proven to be a cost-effective option for treatment of wastewater containing dyes and other pollutants, as it is a simple and low cost technique and does not require high initial investments. The present work aimed to study the adsorption of methylene blue dye (MB) using sugarcane bagasse (SCB). The biomass was characterized by scanning electron microscopy (SEM). Adsorption studies were conducted batchwise. Kinetics, adsorption isotherms, and thermodynamics were studied. The results showed that SCB presented a maximum adsorption capacity of 9.41 mg g^−1^ at 45 °C after 24 h of contact time. Adsorption kinetics data better fitted the pseudo-second order model, indicating a chemical process was involved. The Sips’s three-parameter isotherm model was better for adjusting the data obtained for the adsorption isotherms, indicating a heterogeneous adsorption process. The process showed to be endothermic, spontaneous, and feasible. Therefore, it was concluded that SCB presented as a potential biosorbent material for the treatment of MB-contaminated waters.

## 1. Introduction

With increasing technological development, the world is changing. Consequences of rapid growth include environmental disturbances and pollution problems. In addition to other needs, water demand for industry has increased rapidly and resulted in the generation of a large amount of wastewater containing large amounts of pollutants [1].

Dyes are an important class of industrial pollutants in fields involving paper, leather tanning, food processing, plastics, cosmetics, rubber, printing, dye manufacturing, and textiles. They are organic compounds that have a complex aromatic molecular structure that connect themselves to surfaces to impart color. These structures are present in stable dyes, are very difficult to treat, and have low biodegradability [2].

The discharge of synthetic dye effluent into the environment affects its ecological status, causing several undesirable changes. Highly colored effluents can be very harmful to receiving water bodies, as the dyes have high water solubility even at low concentrations. These compounds are undesirable because they alter the natural appearance of rivers and lakes, in addition to affecting aquatic life, interfering with sunlight transmission, and consequently reducing photosynthesis and oxygenation of water reservoirs [3].

Water body pollution by dyes may be toxic to aquatic organisms, be resistant to natural biological degradation [1], and cause changes in biological cycles. They also pose risks to human health, as studies show that some of these products can be carcinogenic or mutagenic, and cause allergies, dermatitis, and skin irritation [4].

The removal of dyes from wastewater has severe constraints such as high costs, hazardous product formation, and intensive energy requirements. Therefore, the development of efficient, cost-effective, and environmentally friendly technologies is required to reduce dye content in wastewater [5]. Thus, the search for new solutions, and the development of technologies that can cause less environmental damage are necessary. However, removing color from wastewaters through cheaper and environmentally friendly technologies is a major challenge [6].

Biosorption is a subcategory of adsorption where the adsorbent is a biological matrix and can be considered as a technique for the removal of pollutants through a material of biological origin independent of their metabolic activity [7]. The biosorption process has advantages when compared to conventional treatment methods, such as low cost, high efficiency, minimization of chemical utilization, no additional nutrient requirements, biosorbent regeneration capacity, and metal recoverability [8].

A large variety of low-cost adsorbents have been investigated for their ability to remove color from wastewater, such as peat, bentonite, steel-plant slag, fly ash, china clay, maize cob, wood shavings, and silica. However, these low-cost adsorbents have generally low adsorption capacities and need to be used in large amounts. Therefore, there is a need to find new, economical, easily available, and highly effective adsorbents [6].

Sugarcane bagasse (SCB) is the major by-product of the sugar cane industry; it is one of the largest agriculture residues in the world. It is a fibrous residue of sugarcane stalks left over after crushing and extracting sugarcane juice [8]. About 54 million tons of dry SCB are produced annually worldwide and huge amounts of SCB are burned in the fields, resulting in a serious pollution problem. Furthermore, SCB is an abundant, inexpensive, and promising type of industrial waste with a lignin cellulose and polymeric structure (50% cellulose, 25% hemicellulose, and 25% lignin) [9]. Thus, utilization of this agricultural waste as low-cost adsorbent could provide a two-fold advantage with respect to environmental pollution. Firstly, the volume of by-products could be partly reduced, and secondly the low-cost adsorbent could reduce the pollution of wastewaters at reasonable cost [10].

Verifying the availability of the SCB raw material and its characteristics, as well as the demand for alternative wastewater treatment processes, this work aimed to study the removal of methylene blue dye (MB) in aqueous solution using SCB as a low-cost biosorbent.

## 2. Materials and Methods

### 2.1. Collection and Preparation of SCB

SCB was collected in a local sugarcane juice booth in Astorga, PR, Brazil. It was washed with running water to remove dirt and oven-dried at 80 °C for 48 h. Subsequently, it was crushed and sieved using a knife mill Splabor (model SP-030N, Series 99/09), and retained at 20–28, 28–35, and 35–42 mesh to standardize three different particle sizes. The samples were named SBC20, SBC30, and SBC40, respectively.

### 2.2. Characterization of SCB

The superficial morphology of the SCB was characterized by scanning electron microscopy (SEM) under a Shimadzu SS-550 microscope.

### 2.3. Analytical Measurements

Concentration of MB was determined by finding out the absorbance at the characteristic wavelength of 664 nm in the visible region using an UV/vis spectrophotometer. The calibration curve was prepared using a 100 mg L^−1^ standard solution of MB. The calibration plot of absorbance versus concentration on MB showed a linear variation up to 20 mg L^−1^. The amount of dye adsorbed on SCB (*q_e_* in mg/g) was calculated using Equation (1):

(1) $$q_{e}=\frac{\left(C_{0}-C_{e}\right)}{m}V$$

where *C*_0_ (mg/L) is the initial dye concentration in the solution, *C_e_* (mg/L) is the concentration of the remaining dye in contact with SCB, *V* (L) is the volume of the solution, and *m* (g) is the mass of the adsorbent material.

To quantify the remaining MB concentrations, samples were filtered to avoid that SCB particles that remain suspended interfere in the results.

### 2.4. Effect of Particle Size and Adsorbent Concentration

Preliminary adsorption experiments were performed to optimize the SCB mass and particle size. These experiments were conducted batchwise, in duplicate, varying the SCB mass (25, 50, and 100 mg) and the particle size (20, 30, and 40 mesh), with 50 mL of MB solution (5 mg L^−1^) at 25 °C, in an agitated shaker stirred at 150 rpm, and after 24 h samples were withdrawn and the remaining MB concentrations were determined. Variance analysis (ANOVA) and Tukey’s test for mean comparison at 95% reliability (*p* < 0.05) were performed to verify significant differences in the effect of particle size and adsorbent concentration in the removal efficiency using the software OriginPro 8.5 (OriginLab Corporation, Northampton, MA, USA).

### 2.5. Kinetics Adsorption Experiments

For the kinetics adsorption experiments, also performed in duplicate, 100 mg of SCB was added to 50 mL of MB solution (5 mg/L). The mixtures were stirred and after the appropriate time intervals of contact time (15 and 30 min, 1, 2, 3, 4, 6, 8, and 24 h) samples were withdrawn and the remaining MB concentrations were determined.

The results of adsorption kinetics were analyzed according to the main kinetic models: the pseudo first-order and pseudo-second order model, being represented by Equations (2) and (3), respectively.

(2) $$ln\left(q_{e}-q_{t}\right)=\ln\left(q_{e}\right)-K_{1}t$$

(3) $$\frac{1}{q_{t}}=\frac{1}{K_{2}q_{e}^{2}}+\frac{t}{q_{e}}$$

where *q_e_* (mg g^−1^) and *q_t_* (mg g^−1^) are the amount of MB adsorbed at equilibrium and at time *t* (min), respectively, and *K*_1_ (min^−1^) is the rate constant of adsorption of first-order adsorption and *K*_2_ (g mg^−1^ min^−1^) is the kinetic rate constant of pseudo-second-order adsorption process.

### 2.6. Isotherm Adsorption Experiments

To obtain the adsorption isotherms, solutions of different concentrations of MB (1, 2, 5, 8, 10, 15, 20 mg/L) were prepared in deionized water. Volumes of 50 mL of these solutions were added with 100 mg of SCB and stirred at 150 rpm for 24 h. After contact time, the mixtures were withdrawn and the remaining MB concentrations were determined. This procedure was performed in three different temperatures (15 °C, 30 °C and 45 °C). All experiments were performed in duplicate. 

The results obtained from adsorption isotherms were analyzed with respect to the Langmuir and Freundlich [11,12] for two-parameter isotherm models and Sips and Toth [13] for three-parameter isotherm models, represented by Equations (4) to (7), respectively.

(4) $$q_{e}=\frac{K_{L}q_{m}C_{e}}{1+K_{L}C_{e}}$$

(5) $$q_{e}=K_{F}C_{e}^{1/n}$$

(6) $$q_{e}=\frac{q_{m}{\left(K_{s}C_{e}\right)}^{\beta_{s}}}{1+{\left(K_{s}C_{e}\right)}^{\beta_{s}}}$$

(7) $$q_{e}=\frac{q_{m}C_{e}}{{\left(\frac{1}{K_{t}}+C_{e}^{t}\right)}^{1/t}}$$

where *q_e_* is the amount of MB adsorbed at the equilibrium (mg g^−1^), *q_m_* is the maximum adsorption capacity (mg g^−1^), *K_L_* (mg g^−1^) is the Langmuir adsorption equilibrium constant, *K_F_* (L mg^−1^) and *n* are Freundlich constants, *K_s_* (L mg^−1^) and *β_s_* are Sips constants, and *K_t_* (L mg^−1^) and t are Toth constants.

### 2.7. Thermodynamic Parameters

The thermodynamic parameters of adsorption, Gibb’s free energy (Δ*G*°), enthalpy (Δ*H*°) and entropy (Δ*S*°) changes, were calculated by the following equations:

(8) $$∆G{}^{\circ}=-RTlnK_{C}$$

(9) $$lnK_{C}=\frac{∆S{}^{\circ}}{R}-\frac{∆H{}^{\circ}}{RT}$$

where *T* is the absolute temperature (K), *R* is the universal gas constant (8.314 J mol^−1^ K^−1^) and *K_C_* is the thermodynamic equilibrium constant and was calculated by Sips isotherm equation [14].

## 3. Results and Discussion

### 3.1. Charachterization of SCB

**Figure 1:**
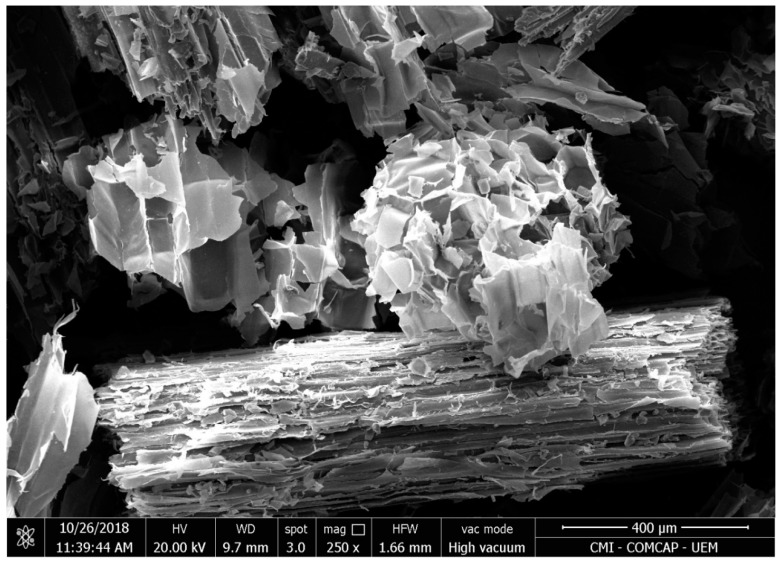
SEM micrograph of general view of the sample of sugarcane bagasse (SCB), showing fibers and residues with 250× magnification.

The characterization of the SCB was performed to determine its superficial morphology, characterized by scanning electron microscopy. Figure 1 presents the micrograph obtained.

SCB morphology is very similar to the internode region of the sugarcane stem. The tissue of the internode is basically formed by vascular bundles, surrounded by sclerenchymatous cells and embedded in parenchyma. Parenchyma cells, which form a soft filling tissue, are roughly separated from the conducting vessels and from the sclerenchyma during the sugarcane milling process. After milling, conducting bundles reinforced by sclerenchyma result in more lignified and resistant sugarcane fibers, while the more fragile tissues constitute the pith residual component [15]. Thus, when observing the SEM micrograph sample of SCB (Figure 1) it is easy to notice two distinct groups: larger particles, called fibers, and smaller particles, called pith. Fibers are compact, rough, and have a thick-walled fiber cells interlinked with the pith. Fibers are constituted by parallel stripes and are superficially covered with extractives. This continuous covering layer is probably composed of hemicellulose and lignin [16,17].

Pores of different size and different shape could be observed. As shown in Figure 1, SCB has irregular structure, which can favor the biosorption of MB on different parts of the biosorbent. These images confirm the previous results obtained by the analysis of the porosity of SCB [18]. 

The characteristics of bagasse depend on several factors, such as the plant species used, the method used to harvest the crop and when it is harvested. SCB is a complex mixture of cellulose, hemicellulose and lignin that makes up the cell walls of the vascular vessels in sugarcane. The fiber content depends on the length and diameter of the stalks, and the number of nodes and distance between nodes. The main elements are silica, potassium, calcium, magnesium, and phosphorus [19].

### 3.2. Effect of Particle Size and Adsorbent Concentration

**Figure 2:**
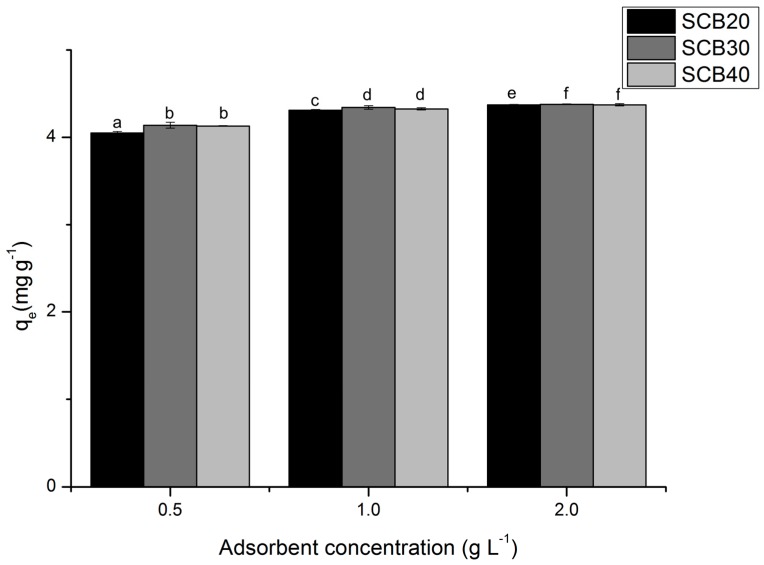
Optimization of sugarcane bagasse (SCB) adsorption under effect of particle size and adsorbent concentration (methylene blue dye (MB) concentration = 5.0 mg L^−1^, adsorbent concentration = 0.5–2.0 g L^−1^, particle size = 20–40 mesh, sample volume = 50 mL, contact time = 24 h, temperature = 25 °C, stirring speed = 150 rpm). Values expressed by the average. Average values followed by the same letter do not statistically differ from one another using the Tukey’s test at 5% significance level.

Adsorption results are presented in Figure 2 varying the particle size (20, 30, and 40 mesh) and adsorbent concentration (0.5, 1, and 2 g/L).

An optimization in MB adsorption was observed under the effect of particle size of SCB. The results using 20 mesh particle size were statistically different from 30 and 40 mesh. However, there was no significant statistical difference in adsorption efficiency when using 30 and 40 mesh particle size.

The increase in SCB concentration improved the adsorption efficiency. The statistical analysis demonstrated that the adsorbent concentration enhances significantly in the adsorption efficiency, thus the concentration of 2 mg L^−1^ resulted in a MB removal of 97.60% and adsorbed amount 4.38 mg/L with 30 mesh particle size. These optimized conditions of adsorption concentration and particle size were used for kinetics and thermodynamic studies.

### 3.3. Kinetics Adsorption Experiments

**Figure 3:**
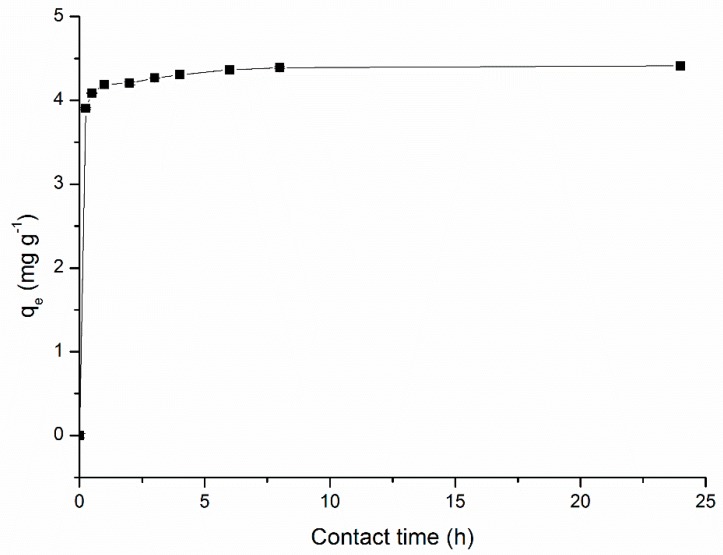
Kinetics adsorption (methylene blue (MB) concentration = 5.0 mg L^−1^, adsorbent concentration = 2.0 g L^−1^, particle size = 30 mesh, sample volume = 50 mL, contact time = 0–24 h, temperature = 25 °C, stirring speed = 150 rpm).

Adsorption equilibrium times differ according to each adsorbent and the compound to be adsorbed. This is a very important factor in adsorption studies, indicating how long it takes the adsorbent to reach maximum adsorption. Figure 3 presents the results obtained in the kinetic study for the adsorption of MB.

According to the obtained results, it was observed that the removal speed was fast and the equilibrium was reached in a short period of time. This could be explained because at the beginning of the adsorption there were many active sites due to the availability of negatively charged active sites on the surface of SCB, such as oxygen groups, carbonyl, alcohol, and phenol groups, ethers, and esters, that interacted with positively charged MB. The rapid adsorption phase was followed by a slow phase adsorption, due to the saturation of adsorbent sites and to electrostatic impediment between positively charged adsorbed species, reducing the adsorption rate [18].

After 3 h of contact time, 95.11% of MB removal was reached. After 3 h of contact time the adsorption achieved 96.74% of the maximum adsorption that was 4.41 mg g^−1^ after 24 h. For better efficiency, the time contact should be increased, but the time increase would not increase considerably the adsorption efficiency. Therefore, it can be said that the adsorption equilibrium was achieved after 3 h. This result is in agreement with the study by Meili et al. (2019) [20], where various agro-industrial residues were used for MB biosorption. They obtained only 90% of MB removal, but using a concentration of MB 20 times higher and a concentration of SCB as adsorbent 2.5 times higher than that used in the present work.

**Figure 4:**
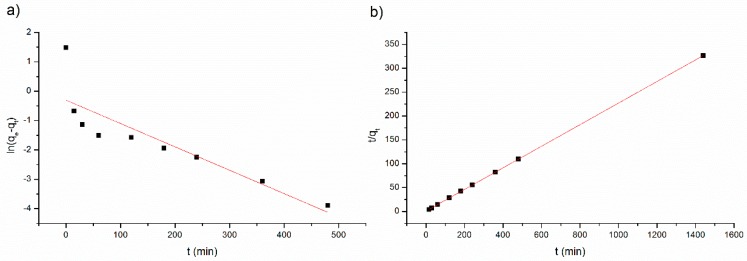
Kinetics models (**a**) the pseudo-first-order model and (**b**) pseudo-second-order model for methylene blue (MB) adsorption onto sugarcane bagasse 30 mesh (SCB30).

The adsorption kinetics data fitted into the kinetics models are shown in Figure 4 and the obtained parameters are given in Table 1.

The amount of MB adsorbed at equilibrium calculated by the first-order model (q_e,cal_ = 0.7365 mg g^−1^, Table 1), obtained from the linear plots (Figure 4a), is not in accordance with the experimental one (q_e_ = 4.41 mg g^−1^). This result shows that the adsorption process may not be reasonably fitted to the first-order equation (R^2^ = 0.7315). The linear plot of t/qt versus t (Figure 4b) for the pseudo-second order model shows a good agreement between experimental and calculated q_e,cal_ value (4.4228 mg g^−1^) presented in Table 1 and also presented a higher correlation coefficient (R^2^ > 0.999). This is agreement with other studies using agricultural waste, since usually the kinetic adsorption data of biosorbents is better represented by a pseudo second-order model for most adsorption systems [2].

Thus, the kinetic model adjustment result indicated the applicability of the pseudo-second-order kinetic model to describe the adsorption of MB onto SCB for the experimental data provides a better fit. Based on the assumption of the successful fitting with the pseudo-second order kinetic model, it can be suggested that chemisorption was the rate-controlling step of adsorption speed control involving valence forces by sharing or exchanging electrons between MB and SCB [21].

### 3.4. Isotherms Adsorption Modeling

**Figure 5:**
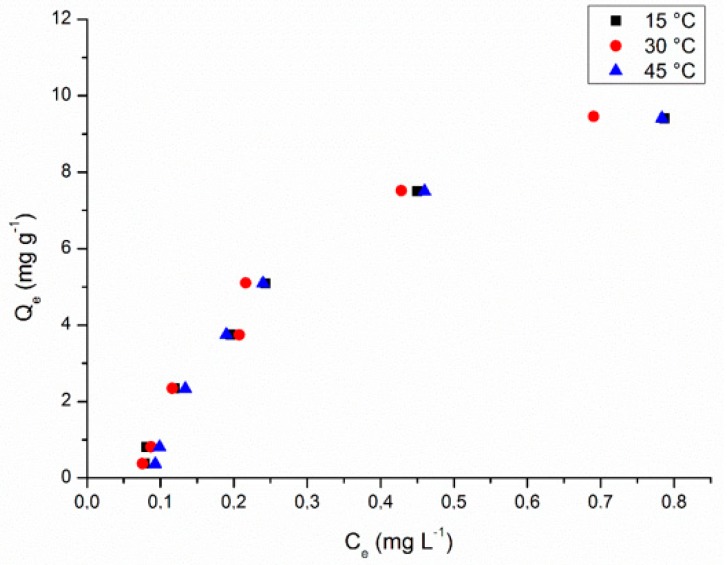
Adsorption isotherms (methylene blue (MB) concentration = 1.0–20.0 mg L^−1^, adsorbent concentration = 2.0 g L^−1^, particle size = 30 mesh, sample volume = 50 mL, contact time = 24 h, temperature = 15–45 °C, stirring speed = 150 rpm).

Adsorption isotherms present the equilibrium relationship between the concentration of adsorbate in solution and the adsorbate retained in the adsorbent at a given temperature and the results are presented in Figure 5.

**Figure 6:**
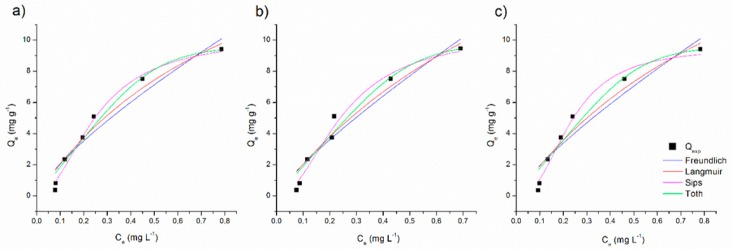
Fitting data for adsorption isotherms models for (**a**) 15 °C, (**b**) 30 °C, and (**c**) 45 °C (methylene blue (MB) concentration = 1.0–20.0 mg L^−1^, adsorbent concentration = 2.0 g L^−1^, particle size = 30 mesh, sample volume = 50 mL, contact time = 24 h, stirring speed = 150 rpm).

At first, it was not possible to observe a notable interference in the adsorption efficiency in relation to the process at different temperatures studied (15–45 °C). To elucidate the effect of the temperature in the biosoption process, the fitting curves for the predicted isotherms models were plotted and are presented in Figure 6. The calculated isotherm parameters are also listed in Table 2.

The Langmuir isotherm model assume that a monolayer adsorption exists on the adsorption surface with a finite number of identical sites, that are energetically equivalent and there is no interaction between the adsorbed molecules. The results for isotherm adsorption data fitting showed that the maximum adsorption capacity (q_m_) was not well predicted by Langmuir model, indicating that this model does not agree with the MB adsorption onto SCB characteristics, and that SCB does not assumes a monolayer adsorption [22].

A reasonable agreement was obtained between the experimental data and the three-parameter models predicted isotherms for MB adsorption. The adsorption data was better fitted by the Sips model (R^2^ > 0.97) than by the Toth model (R^2^ > 0.92), suggesting that MB adsorption on SCB on heterogeneous surface. Sips isotherm is suitable for predicting adsorption on heterogeneous surfaces, thereby avoiding the limitation of increased adsorbate concentration normally associated with the Freundlich model [13]. This result can be confirmed by the morphology characterization, which evidenced an heterogeneous surface (Figure 1).

Although the adsorption capacity obtained in this study was lower compared with some reported values in literature, it is sometimes higher or at least comparable with those of other lignocellulosic biomasses (Table 3). Even though the maximum adsorption value (q_e_) is low for SCB it is still advantageous because it can be used in its natural form without any costly treatment, and after its use, when the adsorbent is saturated it may be used in cogeneration, supplying energy in the industry. All those characteristics make SCB an economical option than other low cost adsorbents.

### 3.5. Thermodynamics Studies

**Figure 7:**
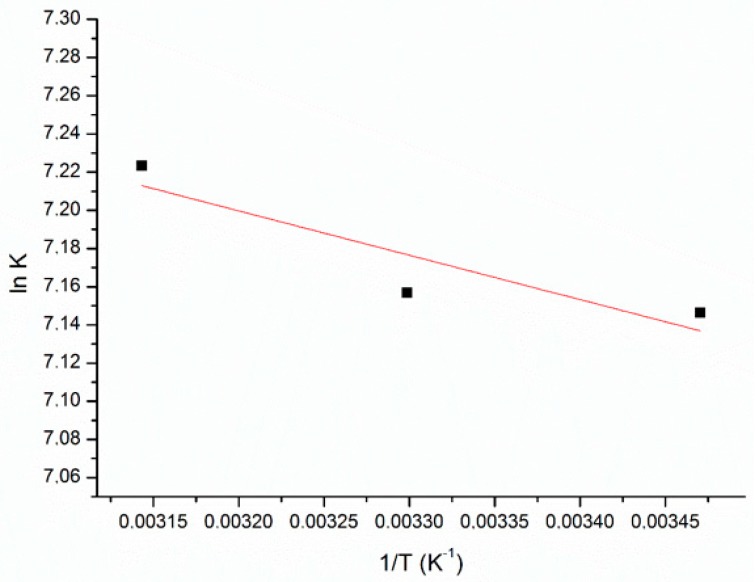
Plot of K_C_ vs 1/T for the estimation of thermodynamic parameters for the adsorption of methylene blue (MB) onto sugarcane bagasse (SCB).

The thermodynamic parameters of adsorption, enthalpy (ΔH°) and entropy (ΔS°) changes, were obtained from the ln K_C_ versus 1/T plot (Figure 7). The Gibbs free energy of the adsorption (ΔG°) was calculated according to the Equation (8) and the results are presented in Table 4.

The results show that the enthalpy of adsorption ΔH° was 1.93 kJ mol^−1^ and entropy change ΔS° was −66 J mol^−1^ K^−1^. The positive value of ΔH° reflects an endothermic adsorption and indicates that the adsorption is slightly favored at higher temperatures. The negatives values of standard free energy change (ΔG°) at all temperatures studied are due to the fact that adsorption process is spontaneous and confirm the feasibility of the process. The decrease in the negative value of ΔG° with an increase in temperature indicates that the adsorption process of MB on SCB becomes more favorable at higher temperatures [29]. The positive entropy states that the MB molecules showed increased randomness at the solid/solution interface during the adsorption on the surface of the SCB, and that some structural changes may occur in on the adsorbent [30].

## 4. Conclusions

It was demonstrated that the utilization of SCB as biosorbent is an eco-friendly technique, as it is a way of minimization of agricultural waste and it also proved to be a promising biosorbent for treating water contaminated with MB. The batch study parameters—contact time, temperature, initial MB concentration, particle size, and SCB concentration—were found to be important parameters in the biosorption processes. The kinetic studies indicated that equilibrium in the biosorption of MB on SCB was reached in 3 h of contact time. The maximum removal capacity of MB was 98.32%. The biosorption kinetics was satisfactorily described using pseudo-second order, suggesting a chemical process for the mechanism of biosorption. SCB has no commercial value and the present study showed that it could be a suitable and economically viable alternative as a good and inexpensive source of biosorbent for wastewater treatment due to its production and high availability, resulting in several environmental benefits.

## Figures and Tables

**Table 1:** Adsorption kinetics models parameters. K_1_ = rate constant of adsorption of first-order adsorption: K_2_ = kinetic rate constant of pseudo-second-order adsorption process; q_e_ = amount of MB adsorbed at equilibrium. R^2^ = Correlation coefficient.

Experimental	Pseudo-First-Order			Pseudo-Second-Order		
q_e_ (mg g^−1^)	K_1_ (min^−1^)	q_e,cal_ (mg g^−1^)	R^2^	K_2_ (g mg^−1^ min^−1^)	q_e,cal_ (mg g^−1^)	R^2^
4.41	0.0080	0.7365	0.7315	0.0518	4.4228	0.9999

**Table 2:** Calculated parameters for isotherm adsorption models. q_m_ is the maximum adsorption capacity; K_L_ is the Langmuir adsorption constant; K_F_ and n are Freundlich constants; K_S_ and β_s_ are Sips constants; K and t are Toth constants.

Model	Parameters	Temperature		
		15 °C	30 °C	45 °C
Experimental	q_m_ (mg g^−1^)	9.4094	9.4575	9.4109
Langmuir	q_m_ (mg g^−1^)	21.7799	27.4314	24.3229
	K_L_ (L mg^−1^)	1.0373	0.8044	0.8626
	R^2^	0.9413	0.9329	0.9163
Freundlich	K_F_ (L mg^−1^)	12.1294	13.6724	12.2655
	1/n	0.7653	0.8268	0.7979
	R^2^	0.9103	0.9128	0.8890
Sips	q_m_ (mg g^−1^)	10.2134	10.4478	9.5644
	K_S_	3.9698	4.0111	4.2878
	β_s_	2.0008	2.0339	2.4233
	R^2^	0.9856	0.9703	0.9798
Toth	q_m_ (mg g^−1^)	9.7364	9.9577	9.7307
	K	16.4588	19.6479	17.2374
	t	4.2907	4.5530	4.6464
	R^2^	0.9547	0.9370	0.9254

**Table 3:** Comparison of biosorption capacities of methylene blue (MB) on various agroindustrial by-products.

Biosorbent	q_m_ (mg g^−1^)	Reference
Betel nut husk	0.32	[23]
Passion fruit peel	2.17	[24]
Banana peel	4.91	[23]
Garlic peel	82.74	[25]
Potato peel	105.26	[26]
Orange peel	157.20	[27]
Watermelon winds	188.68	[28]
Sugarcane bagasse	9.41	This work

**Table 4:** Calculated thermodynamic parameters.

Temperature (°C)	ΔG° (kJ mol^−1^)	ΔH° (kJ mol^−1^)	ΔS° (J mol^−1^ K^−1^)
15	−17.12	1.93	66.04
30	−18.04		
45	−19.11		
